# Surveillance without silos: a One Health data imperative for avian influenza in West Africa

**DOI:** 10.3389/fcimb.2026.1927565

**Published:** 2026-08-26

**Authors:** Emmanuel Musa, Francis Akpa, Godfred O. Boateng

**Affiliations:** 1 Dahdaleh Institute for Global Health Research, York University, Toronto, ON, Canada; 2 Public Health Directorate, Ministry of Health, Lokoja, Kogi State, Nigeria; 3 School of Global Health, York University, Toronto, ON, Canada

**Keywords:** avian influenza (AI), data interoperability, health security, integrated surveillance, One Health, West Africa

## Abstract

Highly pathogenic avian influenza (HPAI) has circulated in West Africa since Nigeria’s first human case in 2006, making the region a key entry point and dispersal hub for multiple virus lineages in sub-Saharan Africa. The 2.3.4.4b virus clade driving the global spread of avian influenza is now infecting mammals more often, a troubling sign that raises the risk of a future pandemic. At the same time, West Africa’s ability to control HPAI is held back by fragmented surveillance systems. Animal, human and environmental health services operate in isolation, rely on incompatible data formats and are managed by separate institutions, which undermines early detection and rapid response. This paper describes the existing surveillance silos in West Africa and outlines the consequences of failing to integrate them, including delayed detection of future human or animal health threats. It highlights emerging examples of One Health surveillance efforts in Nigeria, Senegal, Guinea, Benin and Ghana. From an operational standpoint, five conditions are essential for early warning and timely response: interoperable health information systems; shared governance across sectors; sustainable financing; strong communication with poultry-keeping communities; and effective cross-border collaboration. Establishing fully integrated, system-wide national surveillance platforms is indispensable for effective preparedness. Without such integration, West African countries will continue to detect and respond to emerging health threats too late. Given the region’s recurrent zoonotic spillover and expanding ecological vulnerabilities, embedding data integration at the core of preparedness strategies is essential for mitigating the risk of future pandemics.

## 1 Introduction

Viruses do not respect the administrative boundaries we draw between ministries. A duck carrying an influenza virus can move from a wetland to a backyard flock, then to a live bird market and finally into a household kitchen within days, crossing, in that brief journey, the mandates of multiple government sectors and several professional disciplines. Highly pathogenic avian influenza (HPAI) emerges precisely in these interfaces. However, the surveillance systems designed to detect it remain organized around institutional jurisdictions rather than the ecological realities of the pathogen itself. West Africa has navigated this tension for nearly twenty years. HPAI H5N1 was first detected in Africa in 2006 at a commercial poultry farm in Kaduna State, Nigeria, before spreading to twenty-five of the country’s thirty-six states and the Federal Capital Territory, persisting for nearly two years (Fusaro et al., 2009). During the same period, the virus was detected across the wider West African region, and subsequent phylogenetic analyses confirmed multiple introductions, and the circulation and reassortment of distinct sub-lineages (Cattoli et al., 2009). The threat has never fully receded.

The challenge in West Africa is no longer the absence of surveillance activity (Igboh et al., 2021; Cadmus et al., 2026). Veterinary services routinely test birds (Sulaiman et al., 2021; Meseko et al., 2023); public health programs operate sentinel sites for human influenza (Asante et al., 2022); wildlife and environmental agencies contribute ecological data (Olawuyi et al., 2024). This paper argues that the core challenge is not the absence of surveillance activity, but the fact that these efforts operate side by side rather than in concert (Dsani et al., 2025). They remain divided across separate institutions and systems of data, governance and financing, leaving the most critical early signals to fall between the cracks (Cadmus et al., 2026). Building surveillance without silos is therefore less a scientific hurdle than a systems and policy imperative; one that begins with how data are managed, shared and interpreted.

This article is a narrative synthesis drawing on materials from three main sources: First, peer reviewed studies of avian influenza virology, surveillance and One Health; second, health event reports, guidance and information from Food and Agriculture Organization, World Organization for Animal Health, World Health Organization, Africa Centres for Disease Control and Prevention and national veterinary and public health agencies; and third, the authors’ experience of public health surveillance and of setting up and coordinating One Health network in the West African region. Older sources of foundational information have been included where necessary to describe the origin of an epizootic under scrutiny in recent years but generally the most recent relevant information has been used to construct the narrative. The experience of six countries, namely, Nigeria, Senegal, Guinea, Benin, Burkina Faso and Ghana, is drawn upon to illustrate the evidence base on avian influenza in West Africa for the three dimensions of virology, surveillance and One Health implementation. There are a number of peer-reviewed articles on avian influenza by individuals based in these countries published between 2019 and 2026. The countries identified provide examples from the Anglophone (Ghana and Nigeria) and Francophone (Benin, Guinea and Burkina Faso) countries of the region’s coastal countries as well as those inland in the Sahelian region. Importantly, it is not implied that these countries represent a complete sample of all countries within the Economic Community of West African States (ECOWAS).

Furthermore, this Perspective is not a further restatement of One Health principles whose value is already agreed, but an argument about why avian influenza in particular exposes the cost of leaving them unimplemented. Two features of this pathogen make it an unusually demanding test of integration. Its earliest reliable signal appears in wild birds and the environment, the sector that is everywhere in West Africa, the least funded and least connected, so the component best placed to give the earliest warning is the one most reliably absent when decisions are made. In addition, West African live bird markets are an active reassortment setting, which means the cost of a missed cross-sector signal is not simply a slower response but the risk of a novel genotype emerging unseen. For this pathogen in this region, data integration is both the step that can only occur once coordination is established and the central constraint that determines whether the various components can be effectively combined into a West-Africa-specific early warning architecture.

## 2 A threat that shifts across hosts and borders

Any serious surveillance design has to start from how the virus actually behaves in the setting, because that behavior is what fragmented systems systematically miss. Three features stand out: First, West Africa is not simply a recipient of avian influenza but an active part of its global circulation. A continental analysis of H5 spread found that the region acted as a crucial hotspot for the introduction and onward spread of the virus to other parts of Africa, with both the long-distance poultry trade and wild bird migration contributing to its movement (Fusaro et al., 2019). This matters for surveillance because it means a flock testing positive in one country is rarely an isolated event. It is a node in a network that runs along the East Atlantic flyway and along regional trade corridors, neither of which respects national borders.

Second, live bird markets sit at the center of transmission. Markets bring together birds from large commercial farms, small producers, and scavenging backyard flocks, and they concentrate the traders and intermediaries who move between them. In Nigeria, the single confirmed human case during the early epidemic was linked to contact with poultry in a live bird market, and market surveillance was subsequently established with support from the Food and Agriculture Organization to gauge the threat of human exposure (Waziri et al., 2014). The market is precisely the kind of place where animal, human, and environmental risk converge in one location, and precisely the kind of place that single-sector surveillance struggles to monitor well.

Third, the virus keeps evolving. Since late 2020, global spread of clade 2.3.4.4b viruses has been recorded in West Africa with the first detection in Senegal late 2020, followed by detection in Mali, Nigeria, Niger, and even in local backyard poultry in southern Benin in 2021 (Sanogo et al., 2022). The genomic characterization of these viruses from poultry in southern Benin indicated that they were genetically very similar to those detected in neighboring country Nigeria as well as in several countries in Europe (Lo et al., 2022; Laleye et al., 2022; Meseko et al., 2023). Introduction of these viruses in West Africa apparently was in line with the arrival of Eurasian wild water birds as well as cross-border movement of commercial poultry with neighboring countries (Sanogo et al., 2022). Ongoing surveillance including whole-genome sequencing of human and animal cases in all countries of West Africa is needed for this emerging regional threat. The virus has also reassorted which is a more consequential mechanism because it produces novel genotypes in a single step. H5N1 viruses in three regions of Burkina Faso in 2021 carried a PA gene acquired from zoonotic G1 lineage H9N2 circulating in West Africa, with mutations associated with increased zoonotic potential (Ouoba et al., 2022). The partner in that exchange is not hypothetical. Surveillance in nine Nigerian live bird markets recovered H9N2 from apparently healthy birds carrying the I155T and Q226L substitutions, which shift binding preference toward human-type receptors of the upper airway (Sulaiman et al., 2021). Tracking this requires long-term surveillance and whole-genome sequencing across human and animal populations, coordinated across West Africa (Sanogo et al., 2022).

The stakes of getting this wrong have risen. Clade 2.3.4.4b has driven an unprecedented panzootic, spilling over into a widening range of mammals, including sea lions, mink, cats, and dairy cattle, accompanied by adaptive mutations that support replication in mammalian hosts and by a growing number of human infections (Capelastegui and Goldhill, 2025). The dairy cattle outbreak that began in the United States in 2024 indicates how a virus long treated as an avian and occasional human problem can establish itself in an entirely new mammalian host and spread within it (FAO, WHO and WOAH, 2025). None of this means a pandemic is inevitable. It does mean that the margin for late detection is shrinking, and the mixed human and animal settings that West African markets and smallholdings provide are precisely the conditions that favor viral co-circulation, reassortment, and human exposure. We do not claim these are the most likely sites of emergence globally, a comparative judgment the available evidence cannot support. We argue that they are exactly the settings a fragmented surveillance system is least equipped to watch.

**Table 1** outlines lessons to be drawn from poultry surveillance of emerging diseases during the emergence and spread of clade 2.3.4.4b viruses and correlates these with lessons for cross-sectoral/national surveillance.

**Table 1:** Selected milestones in the trajectory of highly pathogenic avian influenza (HPAI) relevant to West Africa, and their corresponding surveillance implications.

Period and place	Key development	Implication for integrated surveillance	Source
2006, Nigeria	First confirmed African HPAI H5N1 outbreak, in commercial poultry; spread to 25 of 36 states and the Federal Capital Territory over 21 months; one fatal human case.	A national index event with human exposure, indicating the need to link veterinary detection to human-health action from the start.	Fusaro et al., 2009
2006-2008, multiple African countries	Virus reached 11 to 12 African countries; three distinct sub-lineages were introduced, persisted, and reassorted.	Spread is regional, not national; surveillance confined within borders cannot follow lineage movement.	Cattoli et al., 2009
2006 onward, Nigeria	Live bird markets identified as mixing points and probable reservoirs; the only human case was linked to market contact; market surveillance set up with FAO support.	Markets concentrate animal, human, and environmental risk in one place and need jointly owned, not single-sector, monitoring.	Waziri et al., 2014
2006-2019, Africa	West Africa shown to act as a hotspot for introduction and onward spread, driven by both poultry trade and wild bird migration.	Trade and flyway data held by other sectors are essential inputs to animal-health risk assessment.	Fusaro et al., 2019
2020-2021, West Africa	Clade 2.3.4.4b enters the region (Senegal, then Mali, Nigeria, Niger, Benin); Benin isolates linked to Nigeria and to Europe; entry tied to migratory birds and cross-border poultry movement.	Points directly to coordinated, long-term surveillance and whole-genome sequencing across human and animal populations region-wide.	Lo et al., 2022; Sanogo et al., 2022; Laleye et al., 2022
2021-2022 Nigeria and Burkina Faso	467 outbreaks in 31 of Nigeria’s 37 states (H5N1, H5N2, H5N8); Burkina Faso H5N1 acquires PA from G1 lineage H9N2; H9N2 with human-type receptor markers detected in apparently healthy poultry in Nigerian markets.	Reassortment already occurring in West Africa. Its detection requires sequencing linked across countries and across the poultry-human interface.	Meseko et al., 2023; Ouoba et al., 2022; Sulaiman et al., 2021
2021-present, global	Clade 2.3.4.4b drives a panzootic with spillover into mammals, including dairy cattle, alongside mammalian adaptive mutations and rising human cases.	The margin for late detection is shrinking; novel reassortants are likeliest to surface in mixed human and animal settings such as markets and smallholdings.	Capelastegui and Goldhill, 2025; FAO, WHO and WOAH, 2025

HPAI, highly pathogenic avian influenza; FAO, Food and Agriculture Organization of the United Nations; WOAH, World Organisation for Animal Health; IDSR, Integrated Disease Surveillance and Response.

## 3 The fragmentation in the surveillance landscape

If the case for integration is so clear, why do silos persist? In our experience the reasons are structural rather than a matter of goodwill, and they recur from country to country. The first is institutional. Animal health, human health, and the environment usually answer to different ministries, with separate budgets, separate reporting lines, and separate professional cultures. A veterinarian and an epidemiologist may work a few kilometers apart but never share a case definition or report. Regional assessments bear this out. A recent evaluation of Guinea’s One Health platforms using the Africa Centres for Disease Control and Prevention assessment tool found an overall performance score of around 41%, with none of the country’s eight regions reaching the 60% threshold, and with particular weakness in the mobilization of material resources (Bongono et al., 2025). In Ghana, interviews across the human, animal, and wildlife sectors found collaboration proceeding largely through informal personal contact rather than formal mechanisms (Dsani et al., 2025). Coordination structures exist on paper more often than they function in the field (Nana et al., 2022; Bongono et al., 2025).

The second is the data layer itself, which is where fragmentation becomes most damaging. Sectors collect information using different tools, for different purposes, and store it in systems that cannot talk to each other. A recent study of zoonotic surveillance in Senegal identified three mutually reinforcing barriers to data sharing including limited technical and organizational capacity that weakens interoperability and constrains data use; divergent stakeholder attitudes, ranging from genuine commitment to mistrust and reluctance shaped by concerns about transparency and recognition; and fragmented governance, characterized by the absence of a clear regulatory framework, weak coordination and insufficient long-term funding (Diouf et al., 2026). The study also noted that valuable data collected by private actors and non-governmental organizations during routine field activities remain largely unused because no mechanism exists to integrate them. Senegal’s 2021 response is notable. It involved several institutions including the national veterinary laboratory, veterinary services, the national parks directorate, and teams investigating poultry and pelican deaths, working largely in parallel rather than together. They only realized they were dealing with the same event once genome sequencing results were compared (Lo et al., 2022). Data that cannot be combined cannot generate an early signal, however diligently it is gathered (Eneh et al., 2025).

The third is the architecture of the surveillance systems themselves. Across the WHO African Region, countries run Integrated Disease Surveillance and Response (IDSR), which has been designed to take a One Health perspective, and which recognizes that roughly three-quarters of emerging and re-emerging human pathogens originate in animals (WHO Regional Office for Africa, 2019). In practice, IDSR has continued to lean heavily on indicator-based reporting from health facilities, which detects people who are already sick. A review of two decades of IDSR in sub-Saharan Africa concluded that event-based and community-based surveillance, the components most likely to catch an unusual die-off of poultry or wild birds before human cases appear, remain the least developed parts of the system (Mremi et al., 2021). Nigeria’s own One Health prioritization exercise ranked avian influenza second of 36 zoonoses, while noting that the surveillance system meant to track such priorities remains sub-optimal and poorly coordinated across the human and animal health sectors (Ihekweazu et al., 2021). For a pathogen that announces itself first in birds, this is the wrong place to be weakest, but these gaps in the surveillance landscape are persisting across the countries in the region.

## 4 Operational realities of integration

Integration is easier to endorse in policy documents than to achieve it practically in the places where surveillance actually happens (Nana et al., 2022; Dsani et al., 2026). Markets at dawn, wetlands along migratory flyways, and rural households where the first dead bird is noticed, all reveal the same reality. The virus moves across people, species and borders more fluidly than our institutions do. The challenge is not the absence of surveillance, but the fact that existing efforts operate in isolation, producing cracks in the vigilance system that do not deliver effective early warning (Suu-Ire et al., 2025; Cadmus et al., 2026). Creating meaningful integration begins with recognizing these everyday points of disconnect.

At the live bird market, an integrated approach does not simply place a veterinary officer there to swab birds. It links that officer’s findings, almost in real time, to the public health team responsible for the surrounding catchment, so that a cluster of positive birds prompts checks on traders and handlers rather than sitting in a separate veterinary database until the next quarterly report. Market surveillance in Nigeria showed both the value of monitoring these sites and the difficulty of maintaining the connection between detection in animals and protective action for the people exposed to them (Waziri et al., 2014). The technological requirements are modest but the organizational commitment to act on another sector’s data is not.

At the wetland, the same flyways that bring migratory waterfowl to the Inner Niger Delta, the Senegal River basin, and the wetlands of northern Nigeria also bring the viruses those birds carry. Environmental and wildlife surveillance, including sampling wild birds and the water and droppings around roosting sites, offers the earliest warning available, sometimes ahead of the first poultry outbreak. However, it is typically the most poorly funded and least connected component, housed within environment or wildlife agencies that are rarely present when outbreak decisions are made. When the system detects a signal, the key operational issue is how a wildlife or environmental alert is transmitted to the veterinary and public health teams who need to act on it, a step that fragmented systems currently fail to define. The introduction of clade 2.3.4.4b into the region, traced to migratory birds, is a reminder that the environmental sector is not peripheral but central to this work (Sanogo et al., 2022).

In most communities in rural West Africa, the first person to notice dying birds is not an official but a farmer, a trader or a child sent to feed the flock. Community-based surveillance can turn simple observations of unusual events into timely alerts that are acted upon quickly, as long as reporting is easy, trusted and results in visible action. Many unusual events during the 2014–2016 Ebola epidemic were first detected by and reported from within communities. Although these valuable pieces of information were reported, they were not picked up by the disease surveillance systems primarily designed around facilities and laboratories (Abramowitz et al., 2015; Kruk et al., 2015). For avian influenza surveillance and detection, the community to be monitored include people who keep birds, move birds, sell birds and even cook birds, who must all be sensitized and linked to the surveillance system.

Considering cross-border surveillance, poultry and poultry products move across the porous frontiers of the Economic Community of West African States (ECOWAS) in volumes that formal trade statistics do not capture, and the virus moves with them. This is why the genetic links between Benin and Nigerian viruses, are unsurprising because of cross-border circulation (Sanogo et al., 2022), and why a purely national surveillance system is structurally incomplete. ECOWAS has acknowledged this, adopting a regional One Health framework and, through its first regional One Health Zoonotic Disease Prioritisation process in 2018, identifying zoonotic influenza among its seven priority diseases (Goryoka et al., 2021). The framework exists, but the routine, operational exchange of surveillance data across the borders it covers does not yet match it.

## 5 The data imperative

At the center of each of the operational and practical challenges sits the same unresolved question: can the relevant data be brought together quickly enough to act? This is why integration is best understood as a data imperative rather than simply a coordination goal. Coordination meetings, however well attended, do not detect outbreaks (Diouf et al., 2026). Combined, timely, interpretable data does (Ogundiran et al., 2025).

Making that possible requires three elements that are often overlooked in favor of higher-level declarations. The first element is interoperability, which has at least five distinct layers including technical interoperability, the ability of systems to transmit and receive data at all; syntactic interoperability, compatible formats and data structures; semantic interoperability, consistent definitions, coding systems, and interpretation, so that a case means the same thing in each sector; organizational interoperability, aligned workflows, responsibilities, and decision procedures; and legal inter-operability, agreed rules on data access, use, protection, and sharing. Most One Health data-sharing failures in the region are not primarily technical, rather, they arise from semantic, organizational, and legal barriers, which is why simply purchasing compatible software rarely resolves them (Diouf et al., 2026). In countries where the District Health Information System already anchors human health reporting, the practical task is to integrate animal, climate and environmental data to it rather than build a parallel structure that will compete for attention and funding. The second element is data governance, including clear, agreed rules on who may see what, for what purpose and with what protections. The absence of such rules is a major reason field staff hold back information even when they are willing to share it (Diouf et al., 2026). The third element is the discipline of turning data into a product a decision-maker can use including a short, regular, joint risk assessment that reflects animal, human, climate and environmental risk in a single voice, rather than three separate reports no one reads together.

A clarification matters here, because “integration” is easily misread as centralization. We are not proposing a single database into which all sectors pour their records, nor the absorption of animal and environmental data into the human-health platform, an arrangement that would risk entrenching human-health dominance within One Health governance and discouraging the very sectors whose data is hardest to obtain. The more workable model is a federated one: each sector retains ownership of and responsibility for its own system, and the sectors exchange an agreed minimum dataset through interoperable interfaces, feeding a shared layer for joint risk assessment and a regional alert-exchange mechanism. What must be shared is the signal and the agreed minimum data behind it, not the ownership of every record.

**Figure 1:**
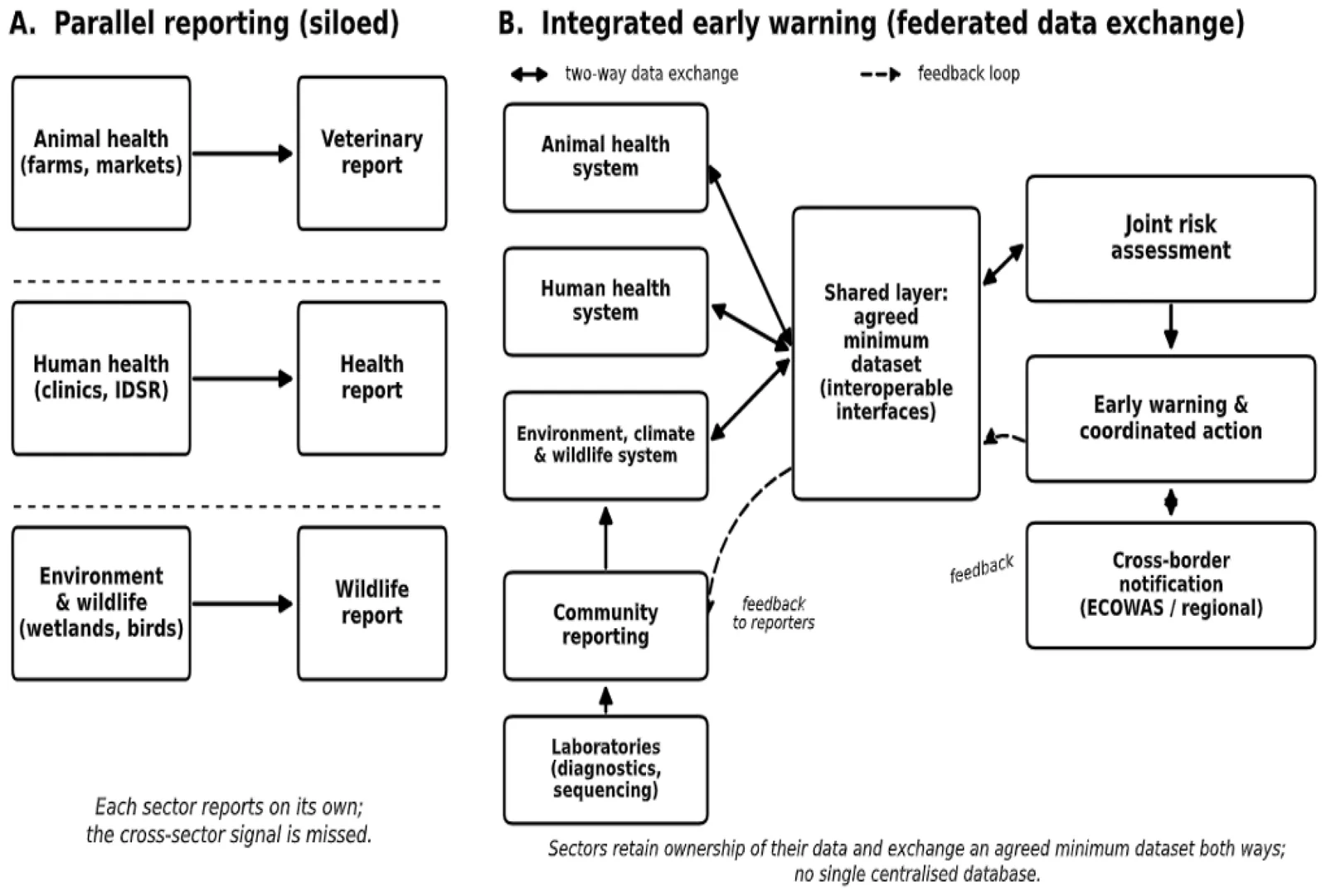
From Parallel reporting to integrated early warning. **(A)** In siloed surveillance, animal, human, and environmental data move along separate reporting chains, so the cross-sector signal that would indicate an emerging avian influenza threat is missed. **(B)** In integrated surveillance, the three streams exchange an agreed minimum dataset through interoperable interfaces (a federated rather than centralized arrangement, in which each sector retains ownership of its own data) to yield a joint risk assessment that drives early warning and coordinated action. IDSR, Integrated Disease Surveillance and Response.

**Figure 1** contrasts the two arrangements described in this paper. In the siloed state, each sector channels its observations into its own report, and the alert that only becomes meaningful when the three are viewed together is never assembled. On the other hand, in an integrated state, the same streams exchange an agreed minimum dataset through interoperable interfaces, feeding a joint risk assessment and, from it, early warning and coordinated action. The difference between the two diagrams is not more data, nor a single system that owns it all. It is whether the data is allowed to meet.

Digital tools and Innovative technology offer a practical opportunity for the shared data layer described in **Figure 1**, provided they are built across sectors rather than within them. Mobile, participatory platforms linking community, animal and human health reporting in near real time are already operational in the region (Kolawole et al., 2026). A smartphone application for example, can allow community reporters and animal and human health officials to submit and act on cases in real time through a single application, with feedback returned to those doing the reporting (Karimuribo et al., 2017). Increasingly, portable genomic sequencing is likewise in routine use in parts of the region and can place a new isolate within its regional lineage in days rather than months (Sanogo et al., 2022). Building on such existing foundations, machine learning could flag unusual patterns across combined veterinary, clinical, climate and environmental streams earlier than any single sector would detect them. Language models can also turn informal event-based signals into structured alerts, but their value in this setting is not yet fully demonstrated and depends on data quality, connectivity, local analytic capacity, data protection, and human validation of every alert (Balakrishnan et al., 2025; Rodrigues et al., 2026). None of these replaces the harder work. An algorithm trained on one sector’s data, governed by no shared rules and running on unreliable connectivity, will reproduce the silos it was meant to dissolve. These tools are only as integrative as the data governance, infrastructure and trust that surround them.

The global policy architecture now supports this direction. The One Health Joint Plan of Action adopted by the Quadripartite organizations; the Food and Agriculture Organization, the World Organization for Animal Health, the World Health Organization and the United Nations Environment Programme, centers its action tracks on zoonotic epidemics and names data systems alongside governance and organizational development as pathways for change (FAO, UNEP, WHO and WOAH, 2022). West African countries are not short of frameworks to align with. The unfinished work is national and local, and it is mostly about structure rather than principle.

## 6 Trust, livelihoods, and the politics of reporting

There is a dimension of integrated surveillance that technical designs routinely underestimate, and that field experience makes impossible to ignore. Surveillance depends on people choosing to report, and that choice is shaped by what they expect to happen next.

Poultry in West Africa is a store of value, a source of protein, and a buffer against economic shocks for millions of smallholder and backyard producers, most of them women. The report of sick birds by these poultry producers is often followed by culling by response officials without prompt, fair, and visible financial compensation. Consequently, the rational response of these producers who depend on those birds is to sell them quickly, move them, or stay silent. A survey of 422 poultry farmers in north-central Nigeria found that exchanging birds between flocks, proximity to water bodies, contact between wild and domestic birds, and seasonal waterfowl migration all significantly influenced H5N1 resurgence, and that socio-cultural and economic behaviors were among the most complex determinants to address (Alhaji et al., 2023). Surveillance then fails not because people do not know what to look for, but because the system has given them a reason not to look or report. The mistrust that surfaces in studies of data sharing among officials (Diouf et al., 2026) has an even sharper counterpart at the level of the household, and it cannot be fixed with a better database.

This is where the systems argument and the policy argument meet. Compensation schemes, support for improved biosecurity that smallholders can actually afford, and transparent communication about why reporting matters are not soft add-ons to surveillance but crucial elements for success. They are part of its core machinery, because they determine whether the earliest alerts ever reach the surveillance system at all. An integrated approach that connects veterinary, public health, climate and environmental data but ignores the livelihoods of the people generating that data will remain blind in exactly the settings where novel viruses are most likely to emerge.

## 7 A practical agenda for surveillance without silos

The following priorities would shift the region from fragmented activity toward genuinely integrated early warning. None is novel on its own. Their value lies in being pursued together, as components of one system rather than separate sectoral projects. The existing diverse streams of data need to be connected before building new structures. The quickest gains come from linking existing animal, human, climate and environmental data through shared standards and a common platform (Suu-Ire et al., 2025), and from producing a regular joint risk assessment owned by all relevant sectors and institutions. A functioning data feed is worth more than another coordination committee (Ogundiran et al., 2025).Event-based and community-based surveillance, and environmental and wild bird monitoring, are the parts of the system most likely to detect avian influenza before it reaches people, and they are currently the weakest (Mremi et al., 2021; Cadmus et al., 2026; Olawuyi et al., 2024). They merit a disproportionate share of new investment rather than the residual. Surveillance platforms should be funded to function, not just to exist. The gap between One Health structures on paper and their performance in practice, evident in the Guinea assessment (Bongono et al., 2025; Dsani et al., 2025), is largely a gap in sustained domestic financing and in the material resources field teams need to operate. Donor projects can initiate this work, but only predictable national budgets can maintain it.

Given the free circulation of the virus across ECOWAS borders, cross-border data sharing and harmonized sequencing should function as routine elements of the regional framework already in place (Goryoka et al., 2021; Laleye et al., 2022; Sanogo et al., 2022), rather than exceptional measures activated only during a declared emergency. Thus, making the entire West African region as the unit of surveillance.

Systems should be built around the people who sustain it. Compensation for culled birds, affordable biosecurity, respectful engagement of poultry producers, and clear data governance collectively will help in ensuring producers and frontline workers trust the system enough to participate (Alhaji et al., 2023; Diouf et al., 2026). Without that trust, even the most sophisticated integration will miss the first case.

**Table 2** shows how these five conditions translate into an operational framework by specifying, for each one, the core function, the responsible institutions, the minimum requirement, and a simple measurable indicator to assess whether it is functioning. The indicators are intentionally straightforward, because a surveillance function that cannot be measured cannot be improved or held accountable.

**Table 2:** An operational framework for integrated avian influenza surveillance in West Africa, mapping five conditions to functions, responsible actors, minimum requirements, and performance indicators.

Condition	Core function and minimum requirement	Responsible actors	Performance indicator
Data interoperability	A standardized minimum dataset and automated cross-sector exchange, using shared case definitions and coding.	Ministries of health and of livestock, reference laboratories, surveillance units.	Proportion of cross-sector alerts exchanged within 24 hours of detection.
Shared governance	Agreed data-access rules, accountability, and predefined response triggers linking one sector’s alert to another’s action.	National One Health platform or coordinating mechanism.	Proportion of priority alerts jointly assessed by the relevant sectors.
Sustainable financing	Domestic funding for routine surveillance operations, not only donor-funded projects.	Ministries of finance and sectoral ministries.	Proportion of recurring surveillance costs financed domestically.
Community trust	Accessible reporting channels, feedback to reporters, and prompt, fair compensation that protects livelihoods.	Veterinary services and local authorities.	Median time from a community report to feedback or compensation; proportion of affected producers compensated.
Cross-border exchange	Routine regional notification and sharing of genomic and epidemiological data.	ECOWAS and national One Health focal points.	Time from laboratory confirmation to regional notification.

## 8 Limitations

The following limitations should be read alongside this Perspective. It is a narrative synthesis, not a systematic or quantitative regional assessment, and the country examples were selected from the available literature rather than sampled comprehensively, so they illustrate rather than represent the region. Evidence availability differs substantially between West African countries. Some countries such as Nigeria and Senegal, have a rich recent peer-reviewed base, while for others the published record is thin, and this unevenness may itself shape which patterns appear prominent. No attempt has been made to quantitatively compare countries’ integrated disease surveillance performance. Furthermore, the operational framework proposed in **Table 2** is not yet operational and will have to be evaluated prospectively. Moreover, because of great differences in the institutional, financial, laboratory and digital capacities among countries in the Region, no single framework is likely to be transferable to another country without important modifications. Finally, the conclusions drawn from the available evidence may also have been influenced by publication and reporting biases, including the greater visibility of outbreaks and programs that attract international funding support.

## 9 Conclusion

Avian influenza in West Africa has, from the outset, been a One Health problem wearing the disguise of a poultry problem. The region’s experience since 2006 shows a virus that moves easily among wild birds, domestic flocks, markets and people, and has now acquired, in other parts of the world, an unsettling ability to establish itself in new mammalian hosts. Surveillance has not kept pace, not for lack of effort, but because it remains fragmented along the very boundaries that are unknown to the virus.

Surveillance without silos is not a slogan about cooperation. It is a concrete and achievable commitment to combine animal, human, climate and environmental data quickly enough to act before an outbreak becomes obvious, to govern that data in ways that earn the trust of the people who generate it, to fund the unglamorous early-warning components properly, and to treat West Africa as the single epidemiological space that it is. The frameworks are already in place. What remains is the harder, less visible work of integration at the level where surveillance actually happens. For a pathogen with pandemic potential, that work is not optional, and it is not something to begin once the next emergency has already arrived. Closing the gap between sectoral data systems is essential for operationalizing One Health approaches, improving early warning capabilities and safeguarding Africa against future pandemic-scale threats.

## Data Availability

The original contributions presented in the study are included in the article/supplementary material. Further inquiries can be directed to the corresponding author.
